# Association between Growth Differentiation Factor-15 and Risk of Cardiovascular Diseases in Patients with Adult Growth Hormone Deficiency

**DOI:** 10.1155/2021/5921863

**Published:** 2021-08-06

**Authors:** Xun Wu, Yunting Wang, Ziyu Ren, Linman Li, Wenjie Qian, Yue Chen, Wei Ren

**Affiliations:** ^1^Department of Endocrinology, The First Affiliated Hospital of Chongqing Medical University, Chongqing, China; ^2^Department of Endocrinology, The Second Affiliated Hospital of Chongqing Medical University, Chongqing, China; ^3^Department of Health Management Center, The First Affiliated Hospital of Chongqing Medical University, Chongqing, China; ^4^General Practice, The 958 Hospital of the People's Liberation Army, Chongqing, China

## Abstract

**Objective:**

Patients with adult growth hormone deficiency (AGHD) confer a heightened risk of cardiovascular disease and increased mortality because of metabolic disorders. Growth differentiation factor-15 (GDF-15) plays an important role in predicting metabolic abnormalities. We sought to investigate the correlation between GDF-15 and cardiovascular risk in AGHD patients.

**Methods:**

The study enrolled 80 AGHD patients and 80 healthy subjects. We analyzed the association between GDF-15 and some major biochemical indicators. The potential association between GDF-15 and cardiovascular disease risk was analyzed.

**Results:**

The AGHD group exhibited increased waist-hip ratio and high-sensitivity C-reactive protein (hs-CRP) and lipid levels compared with the healthy control group. Serum GDF-15 levels in AGHD group were elevated significantly compared with the control group (*P* < 0.001). GDF-15 levels were negatively associated with insulin-like growth factor-1 in AGHD group (*P*=0.006) and positively correlated with waist-to-hip ratio (*P*=0.018), triglycerides (*P*=0.007), and hs-CRP (*P*=0.046). In addition, GDF-15 was positively correlated with Framingham risk score significantly after adjustment for other factors (*r* = 0.497, *P* < 0.001). Moreover, GDF-15 was an independent risk factor for cardiovascular disease in AGHD patients after adjusting for traditional cardiovascular risk factors.

**Conclusion:**

Elevated GDF-15 levels were significantly associated with cardiovascular risk factors and can be considered as a predictive biomarker of cardiovascular risk in AGHD patients.

## 1. Introduction

Growth hormone (GH) affects linear growth in childhood, but GH plays an important role in glucose and lipid metabolism in adulthood mainly by insulin-like growth factor-1 (IGF-1) [1, 2]. Adult growth hormone deficiency (AGHD) is characterized by increased visceral obesity, dyslipidemia, and insulin resistance [3]. Metabolic syndrome is highly prevalent in AGHD, which results in increased risks of cardiovascular disease (CVD) [4–7]. CVD in patients with AGHD may occur earlier than in the ordinary people [8]. AGHD, despite its relative rarity, contributes to increased mortality on account of elevated CVD morbidity [6, 9]. Therefore, identification of early predictive biomarkers of CVD and primary prevention should begin as soon as possible. However, there is no specific biomarker to predict cardiovascular risk of AGHD patients.

Growth differentiation factor-15 (GDF-15), a member of the transformed growth factor-*β* superfamily, is a cytokine that is widely expressed in macrophages, adipocytes, vascular smooth muscle cells, and endothelial cells in response to inflammation and oxidative stress [10, 11]. GDF-15 has been found to be a heart-derived hormone that regulates body growth [12]. More importantly, GDF-15 is also a hormone of regulating glucose and lipid metabolism associated with obesity and diabetes [13, 14]. Recently, GDF-15 was seen as a novel biomarker with high clinical potential because GDF-15 regulates metabolism in addition to inflammation [10, 11]. As a result, GDF-15 is of increasing interest because it can be a useful biomarker. There are increased cardiovascular risk and mortality of AGHD patients, and more accurate predictors like GDF-15 are needed for patients with AGHD. Therefore, it is very important to explore the relationship between GDF-15 and cardiovascular risk in AGHD.

However, no study investigated serum levels of GDF-15 in AGHD patients. In our study, we evaluated the relationship between GDF-15 and cardiovascular risk factors in AGHD patients, aiming to confirm whether GDF-15 can be a biomarker of cardiovascular risk in AGHD. We present new data on the potential of GDF-15 as a predictor of cardiovascular risk in patients with AGHD.

## 2. Materials and Methods

### 2.1. Study Population

In our study, we included 80 AGHD patients at the Endocrinology Department of the First Affiliated Hospital of Chongqing Medical University from September 2018 to December 2020. The AGHD patients (58 females and 22 males; mean age of 42.09 ± 12.98 years; range: 21–74) had not received recombinant human growth hormone (rhGH) treatment before according to medical records. Besides, 80 age- and sex-matched voluntary healthy subjects were included as the healthy control group.

The inclusion criteria for AGHD patients were as follows: (1) small-bodied adults, (2) a history of hypothalamic-pituitary disease (such as Sheehan's syndrome) accompanied AGHD clinical symptoms, and (3) a history of more than one year after pituitary tumor surgery (surgery or radiation therapy). All the patients were diagnosed according to insulin tolerance test (ITT), and patients with GH peak value < 5.0 *μ*g/L were diagnosed with AGHD [15]. This AGHD group included 38 patients with a pituitary adenoma who had been treated with surgery for more than one year, 13 with Sheehan's syndrome, and 20 with pygmyism as well as 9 patients who possibly had idiopathic growth hormone deficiency. Except for growth hormone, AGHD patients had received other pituitary hormonal therapies and the dose had been stable for more than 6 months to ensure that gonadal, thyroid, and glucocorticoid hormones remained in the normal reference range [16].

Exclusion criteria of our study were as follows: (1) a history of diabetes mellitus; (2) hypertension, systolic blood pressure (SBP) more than 140 mmHg, and/or diastolic blood pressure (DBP) more than 90 mmHg; (3) past medical history including coronary heart disease (CHD) or previous cardiovascular events; (4) malignant tumor; (5) hepatic and renal insufficiency; (6) a seizure or mental illness; (7) acute infection; and (8) pregnancy or breastfeeding [17].

Our research was a cross-sectional study approved by the Ethics Committee of the First Affiliated Hospital of Chongqing Medical University, and all study participants provided written informed consent.

### 2.2. Anthropometric and Clinical Parameters of AGHD Patients

A questionnaire was conducted for each AGHD patient including the essential information (height, weight, waist and hip circumference, and blood pressure), the history of smoking, and previous medical history. In addition, hormonal therapies (type, dose, and duration of treatment) were recorded in detail. Height and weight were measured with the patients wearing light clothes and no shoes. Blood pressure was measured three times and the mean value was taken.

Venous blood samples were collected for biochemical analysis (liver and kidney function, lipid levels, and hs-CRP) after fasting for at least 10 hours. Serum was separated and stored at −80°C until evaluation for serum GDF-15 levels by ELISA (Growth Transformation Factor-15 ELISA Kit, CSB-E12009h, Cusabio, Wuhan).

Body mass index (BMI) was calculated using the following formula: BMI = weight (kg)/height (m^2^). The homeostatic model was used to assess the insulin resistance index: HOMA-IR = fasting insulin (mU/L) × (fasting serum glucose (mmol/L)/22.5) [18]. Framingham risk score (FRS) was calculated for each patient according to blood pressure, serum lipid levels, and smoking history. The 10-year risk scores for CVD were calculated based on the cohort risk assessment equations [19].

### 2.3. Statistical Analysis

We used statistical analysis software (SPSS, version 22.0, IBM, Armonk, NY, USA) to analyze all kinds of clinical data. Quantitative variables were expressed as the means and standard deviation (SD) or median and interquartile range (IQR). The normal distribution of the data was tested by one simple Kolmogorov–Smirnov test. Student's *t*-test was used for normally distributed continuous variables, and the Mann-Whitney *U* test was used for nonnormally-distributed continuous variables. The relations of GDF-15 with other demographic and laboratory characteristics were evaluated by Pearson, Spearman correlation analysis, and multiple linear regression. In order to determine whether GDF-15 was an independent cardiovascular risk factor of AGHD patients, we used chi-square test and binary logistic regression analyses and expressed the results as odds ratios (OR) and their 95% confidence intervals (CIs). *P* values less than 0.05 (*P* < 0.05) were considered statistically significant for all analyses.

## 3. Results

### 3.1. Demographic Data

The characteristics of the control group and AGHD patients are shown in Table 1. The average age of AGHD patients was 42.09 ± 12.98 years, and that of the control group was 43.53 ± 9.98 years (*P*=0.314). There was no difference in the blood pressure, waist circumference, and hip circumference between the two groups. But waist-hip ratio (WHR) associated with abdominal obesity increased in AGHD group (0.81 ± 0.06 versus 0.86 ± 0.06, *P*=0.003). BMI, triglycerides (TG), and low-density lipoprotein cholesterol (LDL-C) also increased in the AGHD patients, although there were no statistical differences in our study. However, high-density lipoprotein cholesterol (HDL-C) was lower in AGHD group (1.49 (1.28–1.79) versus 1.39 (1.13–1.55), *P*=0.008). Compared with the control group, hs-CRP levels were higher significantly in AGHD group (0.70 (0.26–1.16) versus 1.63 (0.75–1.83) mg/L, *P* < 0.001).

### 3.2. Levels of Serum GDF-15

**Figure 1:**
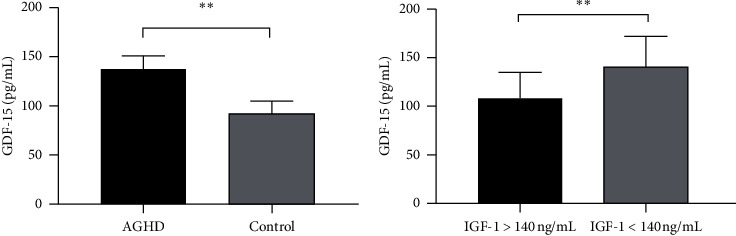
The serum levels of GDF-15. (a) Serum GDF-15 levels of AGHD increased; (b) serum GDF-15 levels increased significantly in the subgroup with IGF-1 < 140 ng/mL of AGHD patients (^∗∗^*P* < 0.01).

Mann-Whitney *U* test was used to investigate the expression of GDF-15 in the control and AGHD groups. Serum GDF-15 levels were significantly higher in AGHD group as compared with the control group (132.38 (97.05–183.16) pg/mL versus 90.45 (61.09–131.90) pg/mL, *P* < 0.001; Figure 1(a)). We stratified the 80 AGHD patients into two subgroups according to 140 ng/mL of serum IGF-1 levels, because people with IGF-1<140 ng/mL had 5-fold higher cardiovascular risk than people with IGF-1>140 ng/mL [20]. We found that GDF-15 levels increased in the subgroup (IGF-1<140 ng/mL) (107.90 (68.86–151.01) versus 140.93 (113.08–229.24), *P*=0.003; Figure 1(b)).

### 3.3. Association between GDF-15 and Cardiovascular Risk in AGHD

**Figure 2:**
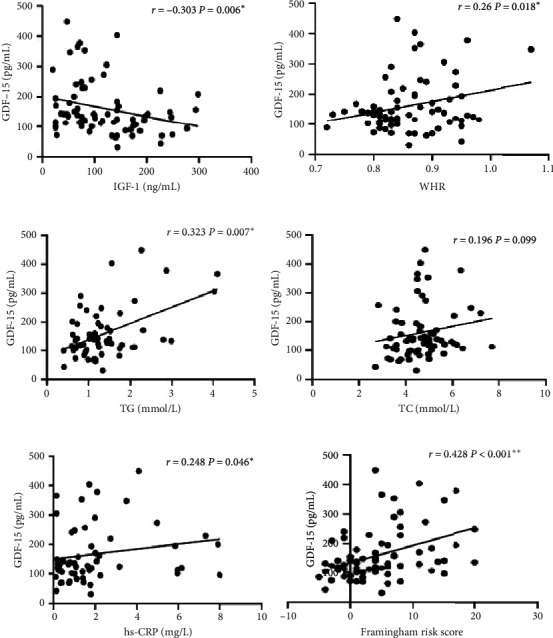
Correlations between GDF-15 and other indicators. GDF-15 was negatively correlated with (a) IGF-1 (*r* = −0.303, *P*=0.006). GDF-15 was positively correlated with (b) WHR (*r* = 0.26, *P*=0.018), (c) TG (*r* = 0.323, *P*=0.007), (d) TC (*r* = 0.196, *P*=0.099), (e) hs-CRP (*r* = 0.248, *P*=0.046), and (f) FRS (*r* = 0.428, *P* < 0.001) (^*∗*^*P* < 0.05; ^∗∗^*P* < 0.001).

In order to explore whether GDF-15 was related to other cardiovascular risk factors of AGHD patients, Pearson or Spearman correlation analysis and multiple linear regression were used. Firstly, GDF-15 was negatively correlated with IGF-1 (*r* = −0.303, *P*=0.006; Figure 2(a)), while GDF-15 was positively correlated with WHR (*r* = 0.26, *P*=0.018; Figure 2(b)), TG (*r* = 0.323, *P*=0.007; Figure 2(c)), TC (*r* = 0.196, *P*=0.099; Figure 2(d)), hs-CRP (*r* = 0.248, *P*=0.046; Figure 2(e)), and FRS (*r* = 0.428, *P* < 0.001; Figure 2(f)). Moreover, GDF-15 was positively correlated with FRS significantly after adjustment for HDL-C, HOMA-IR, LDL-C, and hs-CRP which were the cardiovascular risk factors (*r* = 0.497, *P* < 0.001).

In addition, we divided the 80 AGHD patients into four subgroups according to the GDF-15 quartiles, and the quartiles ranges were Q1: <104.25 pg/mL, Q2: 104.25 to 137.45 pg/mL, Q3: 137.45 to 199.69 pg/mL, and Q4: >199.69 pg/mL. We found that the higher serum level of GDF-15, the greater the risk of cardiovascular disease by chi-square test (Q1: 40%, Q2: 65%, Q3: 70%, and Q4: 85%, *P*=0.024). In order to explore whether GDF-15 predicts cardiovascular risk in young and middle-aged people, we excluded AGHD patients above 60 years of age because CVD had earlier onset in AGHD patients compared with general population [3, 8]. GDF-15 was an independent risk factor for cardiovascular disease in AGHD patients below 60 years of age after adjusting for other cardiovascular risk factors such as BMI, HOMA-IR, LDL-C, and hs-CRP by binary logistic regression (*P* < 0.05). The study suggested that GDF-15 was an independent risk factor for CVD in AGHD (Table 2).

## 4. Discussion

Cardiovascular complications occurring early significantly increase the mortality of AGHD patients [3, 20]. However, there is no specific biomarker to predict cardiovascular risk of AGHD patients at present. Our study found that GDF-15 had the potential to be a cardiovascular predictor in patients with AGHD. To our knowledge, this is the first study to investigate the relationship between increased serum GDF-15 levels and cardiovascular risk in AGHD patients.

### 4.1. Association between Serum GDF-15 Levels and GH/IGF-1 Axis

Because low GH secretion is linked to obesity [21], abdominal adiposity is a typical clinical feature for AGHD [22]. We found that serum GDF-15 levels increased in AGHD patients as it did in obese people compared with the control group [23]. In addition to this, GDF-15 was positively correlated with WHR, which was associated with abdominal obesity in our study.

GH/IGF-1 axis plays a significant role in AGHD patients who are in low serum level of IGF-1 [1, 3]. Wang et al.'s study showed a negative correlation between GDF-15 and IGF-1 in children, and our study found a similar result in AGHD [12]. Serum IGF-1 level was significantly lower in subjects with acute coronary syndrome and early-onset CVD. Besides, it was associated with increased mortality [24]. The patients with lower IGF-1 level are at a higher cardiovascular risk [20], because IGF-1 has an antiatherosclerosis effect by improving endothelial cell function [25]. GDF-15 can be upregulated in endothelial cells and reflect endothelial activation, which is an important progression of atherosclerosis [9, 13]. This could possibly explain the negative correlation between GDF-15 and IGF-1 found in our study. These results demonstrated that GH/IGF-1 axis may have an association with GDF-15 in some way, which needs to be explored by experiment in further studies.

### 4.2. Association between GDF-15 and Cardiovascular Risk Factors

AGHD patients suffer from dyslipidemia besides abdominal obesity, because GH plays a major role in metabolism, especially lipolytic effect on visceral adipose tissue in adulthood [3, 26]. Moreover, dyslipidemia is the strongest contributor of the excess of cardiovascular risk in AGHD [3]. Our findings suggested that GDF-15 was positively correlated with TG in AGHD. Not only are cardiovascular complications of AGHD patients related to dyslipidemia, but also they are associated with the increased intima-media thickness and vascular endothelial dysfunction [3, 27]. The emergence of the foam cells in the arterial intima is considered to be one of the earliest manifestations of atherosclerosis, referring to the preclinical atherogenesis, and macrophages are now considered to be significant in the process [28]. GDF-15 can be induced by saturated fatty acids in macrophages which can damage the vascular system by inducing lipotoxicity [29, 30]. Moreover, GDF-15 can lead to endothelial dysfunction through activation of reactive oxygen species pathway [31]. These results suggested that GDF-15 may be more sensitive than other indicators at an early stage for predicting cardiovascular risk of AGHD patients.

Besides lipid metabolism and arterial endothelial cell dysfunction, AGHD is also closely related to chronic inflammatory conditions [32]. Systemic inflammation has been confirmed to be linked to the development of CVD by numerous studies recently [33]. Chronic inflammation can result in oxidative stress (OS), which is the important cause of cardiovascular complications in AGHD [34–36]. Since the vascular endothelium is very sensitive to OS, a consequence of OS is endothelium barrier dysfunction [37]. Inflammation and OS markers can decrease after GH replacement therapy of AGHD patients, because GH exerted an antioxidative stress effect [26]; hs-CRP is the well-known cardiovascular risk factor [34, 38]. Our study showed that hs-CRP was significantly higher in the AGHD group than in the controls, and GDF-15 was associated with hs-CRP in AGHD.

Moreover, endoplasmic reticulum (ER) stress-initiated inflammation can lead to the progression and pathogenesis of cardiovascular diseases which will be a novel therapeutic target in recent studies [39]. GDF-15 can be induced by ER stress and involved in atherosclerotic lesion progression by regulating cells death and inflammatory responses to vascular injury. In addition, development of atherosclerotic lesions is paralleled by a significant increase in GDF-15 levels [40]. However, GDF-15 deficiency protects against atherosclerosis by attenuating C-motif chemokine receptor 2-mediated macrophage chemotaxis [41]. Thus, our study indicating that GDF-15 may reflect cardiovascular risk in AGHD is supported by previous findings.

As we all know, FRS was an effective predictor of cardiovascular risk, associated with decreased mitochondrial oxidative capacity. Mitochondria could represent an important target for intervention in cardiovascular health [42]. On the one hand, GDF-15 was related to mitochondrial dysfunction closely [43, 44]. On the other hand, GDF-15 showed a linear correlation with FRS after adjusting for other cardiovascular risk factors. Even when adjusting for other variables in logistic regression analysis, GDF-15 remained as an independent cardiovascular risk factor of AGHD in our study. Taken together, our study speculated that GDF-15 could be a cardiovascular biomarker of AGHD.

However, there are some limitations in our study. Firstly, the causal relationship between GDF-15 and cardiovascular risk cannot be determined because of the cross-sectional research study. Another limitation is that we need to expand the sample size and prospective data to confirm whether GDF-15 is also potentially associated with other cardiovascular risk factors in AGHD. We believe that our results can provide more references for future large-scale research.

## 5. Conclusions

In conclusion, we observed that serum GDF-15 levels increased in AGHD patients, and GDF-15 was negatively correlated with IGF-1. In addition, elevated levels of GDF-15 were significantly associated with cardiovascular risk factors. We provided evidence that GDF-15 can be considered as a biomarker for predicting cardiovascular risk in AGHD.

## Figures and Tables

**Table 1:** Baseline features of the AGHD patients and the healthy control group.

Variables	Control	AGHD	*P* value
Age, *y*	43.53 ± 9.98	42.09 ± 12.98	0.314
BMI, kg/m^2^	22.24 ± 2.57	22.94 ± 5.47	0.291
WC, cm	76.34 ± 8.55	79.45 ± 9.44	0.063
HC, cm	92.43 ± 5.83	92.07 ± 6.90	0.863
WHR	0.81 ± 0.06	0.86 ± 0.06	0.003^*∗*^
SBP, mmHg	114.67 ± 13.55	114.09 ± 13.77	0.554
DBP, mmHg	71.06 ± 9.22	73.44 ± 10.62	0.193
ALT, U/L	18 (13–32)	23 (15–29)	0.268
AST, U/L	19 (16–24)	23 (17–25)	0.003^*∗*^
Cr, umol/L	61 (53–70)	61 (58–71)	0.302
FPG, mmol/L	5.2 (4.9–5.5)	4.9 (4.6–5.3)	0.008^*∗*^
TC, mmol/L	4.54 (4.05–4.93)	4.50 (4.14–5.06)	0.173
TG, mmol/L	1.03 (0.84–1.52)	1.25 (0.89–1.54)	0.112
HDL-C, mmol/L	1.49 (1.28–1.79)	1.39 (1.13–1.55)	0.008^*∗*^
LDL-C, mmol/L	2.52 (2.14–3.12)	2.71 (2.24–3.36)	0.064
hs-CRP, mg/L	0.70 (0.26–1.16)	1.63 (0.75–1.83)	<0.001^∗∗^

Data were mean ± SD or median (IQR). BMI = body mass index; WC = waist circumference; HC = hip circumference; WHR = waist-hip ratio; SBP = systolic blood pressure; DBP = diastolic blood pressure; TC = total cholesterol; TG = triglycerides; HDL-C = high-density lipoprotein cholesterol; LDL-C = low-density lipoprotein cholesterol; hs-CRP = high-sensitivity C-reactive protein; AST = aspartate aminotransferase; ALT = alanine aminotransferase; Cr = creatinine; IGF-1 = insulin-like growth factor-1. ^*∗*^*P* < 0.05 and ^∗∗^*P* < 0.001.

**Table 2:** Multivariate logistic regression analysis.

Factors	Model 1		Model 2		Model 3	
	OR (95% CI)	*P* value	OR (95% CI)	*P* value	OR (95% CI)	*P* value
Gender	0.576 (0.159–2.084)	0.400	0.524 (0.139–1.979)	0.341	0.528 (0.125–2.229)	0.385
Age	1.038 (0.981–1.098)	0.200	1.033 (0.976–1.094)	0.261	1.033 (0.972–1.099)	0.296
GDF-15^#^	1.013 (1.002–1.025)	0.022^*∗*^	1.012 (1.001–1.023)	0.034^*∗*^	1.012 (1.001–1.024)	0.038^*∗*^
HOMA-IR	1.100 (0.853–1.419)	0.462	1.099 (0.854–1.416)	0.463	1.202 (0.816–1.771)	0.351
LDL-C	1.072 (0.562–2.044)	0.833	1.101 (0.557–2.179)	0.782	1.223 (0.599–2.497)	0.580
Hs-CRP	—	—	1.344 (0.876–2.061)	0.176	1.364 (0.843–2.207)	0.207
BMI	—	—	—	—	0.845 (0.672–1.062)	0.148

^#^GDF-15 was an independent risk factor for cardiovascular risk after adjusting for other factors among AGHD patients who were below 60 years of age. ^*∗*^*P* < 0.05.

## Data Availability

The data that support the findings of this study are available from the corresponding author upon request.
